# Movement Sequence Learning: Cognitive Processing Demands to Develop a Response Structure

**DOI:** 10.5334/joc.128

**Published:** 2021-02-04

**Authors:** Christina Pfeifer, Julia Harenz, Charles H. Shea, Stefan Panzer

**Affiliations:** 1 Saarland University, Im Stadtwald B8.2, D-66041 Saarbrücken, Germany; 2 Department of Health and Kinesiology, Texas A&M University, US; 3 Texas A&M University, US

**Keywords:** Cognitive Control, Learning, Response speed

## Abstract

An experiment was designed to investigate the impact of a dual-task on the response structure of a 16-element movement sequence. The primary task was to move a lever to targets sequentially presented horizontally on the screen by elbow extension/flexion movements. The secondary task was a simple reaction time task triggered by moving the lever through targets at the middle and the end of the sequence. Participants were permitted to acquire the movement sequence on one day, and to perform the sequence on a second day under single-task and dual-task conditions. The results of the acquisition phase indicated that participants increased their performance over practice. Day 2 analysis indicated that performance of the repeated sequence was not deteriorated by the dual-task. This finding indicated that the response structure of the movement sequence performance was stable with regard to the secondary task. The current results are partially consistent with the theoretical assumption of an abstract representation for movement sequence execution.

## INTRODUCTION

When performing a movement sequence for the first time the sequence is executed slowly and the movement is not produced very fluidly (Ramkumar, Acuna, Berniker, Grafton, Turner, & Kording 2016; Muehlbauer, Panzer, & Shea, 2007). Each element of the sequence is executed in a discrete manner. As the sequence is practiced the time required to move from one element to the next (element duration) is further reduced. With additional practice, participants become less reactive to the visually presented elements, because they can anticipate the upcoming element in the sequence (Vieluf, Massing, Blandin, Leinen, & Panzer, 2015). This sequence knowledge results in an increasingly more rapid, continuous and fluid production of the sequence (Clegg, DiGirolamo, & Keele, 1998; Shea, Kovacs, & Panzer, 2011; for reviews). One prominent theoretical explanation for the decreased element duration is that during practice participants started to “chunk” or “package” (Verwey, 1999; Sakai et al., 2003) two or more individual elements in a movement sequence together. This suggests that groups of elements are executed as relatively independent subsequences (Muehlbauer et al., 2007). Generally, a subsequence is characterized by a relatively long movement time to the first element followed by relatively short movement times to one or more of the following elements (Povel & Collard, 1982). The delay prior to the first element in a subsequence was thought to occur because the subsequence had to be retrieved, programmed, and/or otherwise readied for execution. The following elements in the subsequence are produced more rapidly than the first element because processing related to their production was completed during the processing of the first element in the subsequence (e.g., Clegg et al., 1998). With extended practice, performers do not form larger subsequences, but they do create a more seamless transition between subsequences minimizing subsequence-to-subsequence processing delays (Park & Shea, 2005). The independent subsequences are concatenated so that the larger overall movement sequence response structure emerged, and the sequence is executed more fluidly (e.g. Braden, Panzer, & Shea, 2008). Recent research demonstrated that a sequence with a developed response structure can be transferred to an un-practiced set of effectors (Park & Shea, 2005), rescaled in amplitude (Wilde & Shea, 2006) and forces (Muehlbauer et al., 2007) without a loss of performance as long as the changes are proportional across the entire sequence. These findings let the authors conclude that the sequence structure is represented in a relatively abstract way (Shea et al., 2011).

The purpose of the present experiment was to determine whether individuals after learning a movement sequence can also effectively perform the sequence in a dual-task situation. This finding would provide evidence about the stability of the represented movement sequence structure. Theoretical perspectives proposed that abstract representations are primarily responsible for sequence execution in the early stages of learning, and at this stage of learning sequence execution requires additional processing demands and attention (Clegg et al., 1998; Hikosaka et al., 1999). If the response structure is stored in an abstract manner, as is suggested in recent research, one would expect that the presentation of a dual-task during sequence execution would result in increased element duration, because the response structure essentially determines the speed with which the subsequences are processed and executed (Park & Shea, 2005). We also tested participants with random sequences under a single-task and a dual-task condition. The random tests provide references from which to determine general learning effects from those related to the specific sequence used during the acquisition phase of the experiment.

## METHODS

### PARTICIPANTS

Undergraduate students (N=16) participated in the experiment for course credit. The number of participants was calculated by G*Power (Faul et al., 2007) using the power of 80% and the effect size ‘f’ = .55 from the Vieluf et al., (2015) experiment. The participants had no prior experience with the experimental tasks and were not aware of the specific purpose of the study. All participants were right-hand dominant as determined by the Edinburgh Handedness Inventory (Oldfield, 1971) completed prior to the experiment. Informed consent was obtained prior to participation in the experiment.

### APPARATUS

**Figure 1:**
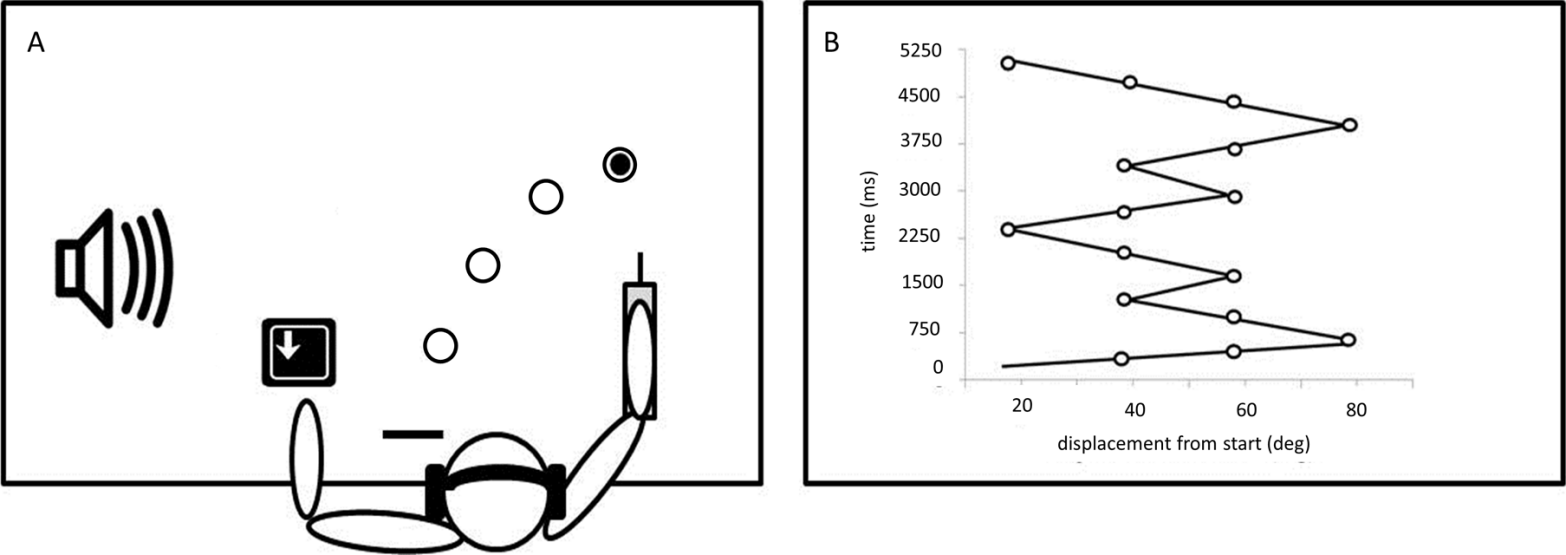
Schematic illustration of the experimental setup. The apparatus (1A) with a participant sitting in front of the video screen and the projected targets, wearing a headphone, and the response box with the arrow, and the sequence pattern (1B).

The apparatus consisted of a horizontal lever (42 cm long) affixed at the proximal end to a near frictionless vertical axle (see ***Figure 1***). The lever was positioned on the right side of the table and was used for right arm movements. The axle of the lever, which rotated freely in ball-bearing supports, allowed the lever to move in the horizontal plane over the table surface. Near the distal end of the lever, a vertical handle was attached. The position of the handle could be adjusted so that, when the participant rested their forearm on the lever, their elbow aligned over the axis of rotation, they could comfortably grasp the handle (palm vertical). The horizontal movement of the lever was monitored (1000 Hz) by a potentiometer that was attached to the lower end of the axle. The potentiometer data were used to provide the lever position information to the participant and stored for later analysis. The experiment was programmed with Matlab R2019a software from MathWorks© (The MathWorks, Inc., Natick, MA). The targets and total movement time were projected on a 48’’ video screen (temporal resolution 75 Hz; spatial resolution 1920 × 1080) positioned in a horizontal plane on a table.

For the secondary task, auditory stimuli were presented by a noise canceling over–the-ears headset (Sennheiser PC 320). Reaction times were collected with a response box (USB connected Arduino microcontroller) with a marked key. To reduce the interaction with other software that affects the timing of the computer all software and supports running in the background of the computer were closed and all other not used USB devices were disconnected.

### EXPERIMENTAL GROUP, TASK AND PROCEDURE

After entering laboratory, the participants were seated in a chair facing the table and the apparatus which was adjusted so that the participants’ lower arm was at approximately an 80-degree angle to the upper arm at the starting position. Instructions were presented on the video screen informing participants of how to perform the two tasks. To begin each trial participants were told to move the lever to the starting position (designated as 0 degrees). When the start position was achieved, the outlines of four circles (targets) were projected on the video screen (***Figure 1A***). The diameter of the target represented 2 degrees of elbow extension (or flexion) with the centers of the targets representing lever positions of 20°, 40°, 60°, and 80° degrees from the start position. The flexion/extension sequence-task consisted of 16 elements, with the target order of the lever position in degree 40°, 60°, 80°, 60°, 40°, 60°, 40°, 20°, 40°, 60°, 40°, 60°, 80°, 60°, 40°, and 20°. An illustration of the target positions and movement pattern required by the sequence is provided in ***Figure 1B***. After a random foreperiod (2-5 s in .5 s intervals) a start tone (1000 Hz for 100 ms) was presented and the first target (40°) was illuminated (outline was filled in). Thus, the presentation of the target positions served as a warning that the trial was about to begin and illumination of the first target provided the cue to begin the movement sequence. The sequence-task was the primary task. For the sequence-task, upon crossing the edge of the illuminated target the illumination was “turned off” and the next target in the sequence was immediately illuminated until the sequence was completed. Participants were instructed to move the lever from one illuminated target to the next as quickly and smoothly as possible. If the participant missed a target, the target remained illuminated until the participant returned the lever to the target position. The participant was not required to dwell at the target position. Simply passing into the target area was sufficient to achieve the target. When the participant had completed all 16 elements of the sequence, a stop tone was presented and the display of the targets was removed. Following a 5 s delay the time for the movement sequence-task was presented as knowledge of results (KR). However, to ensure that participants performed the task in a smooth and continuous manner they were informed they had to achieve a sequence-task time of at least 3 to 4 s for the 16 elements. Note, participants were allowed to perform the movement sequence-task faster, but they were encouraged to follow the exact sequence pattern. After a rest interval of five seconds, the next trial started. Participants were instructed to perform the sequence rapidly and as accurately as possible. A total of 150 trials were provided during acquisition. Note, during acquisition the participants performed one sequence in a repeated manner. Ten trials of the 16-element sequence were averaged in each block. Retention and three transfer tests (same sequence [repeated] were administered under dual-task conditions, a random sequence under single-task, and a random sequence under dual-task; order was counterbalanced) were conducted 24 hrs after the completion of the acquisition session. The participants performed a retention test under the same condition as experienced during acquisition except that KR was not provided. For the second task in the dual-tasks tests, the participants were instructed to react to two auditory stimuli presented during sequence execution by pressing the marked key on the response box as fast as possible with their left middle finger. In the dual-tasks tests, participants were instructed to perform the two tasks with equal priority. Note, the middle finger rested on the key. To identify the effect of a secondary task on the primary task and not to disperse this effect across the continuous movement sequence by randomly triggering the secondary task, the temporal placement of the auditory stimuli was at the 2^nd^ quarter (Element 7) and at the end 3^rd^ quarter (Element 12) of the 16-element sequence. In each test on Day 2, participants were instructed to perform 15 trials. The 15 trials were averaged in each block. To increase uncertainty, 20% catch-trials without a dual-task were included randomly. The random sequence involved the same number of elements but the order was different. Note, the random sequences were derived in a quasi-random way with the limitations that elements would not be repeated, the number of required reversal points were the same, and the total distance moved in the repeated and the random sequence was the same. In addition, different random sequences were used in each block.

### DATA ANALYSIS AND STATISTICS

Data analysis was performed using Matlab (Mathworks, Natick, MA, R2019a). The individual trial time series were used to compute lever displacement, velocity, and acceleration. To reduce noise in the data, the angular displacement time series was filtered with a low-pass filter (2^nd^ order dual-pass Butterworth filter) with a cutoff- frequency of 10 Hz. A 3-point difference-algorithm was used to compute the velocity signal. The velocity signal was smoothed with a mobile 3-point average algorithm before computing angular acceleration. Element duration was computed as an average of the 16 elements of the elapsed time from “hitting” (crossing the target boundary) the currently illuminated target to “hitting” the next illuminated target. The number of zero crossings on the acceleration record were also determined. To determine the number of zero crossings of the acceleration trace the second derivation from the displacement was calculated and the number of zero crossings of the acceleration record was enumerated and averaged across a block. Previous research has shown that zero crossings in the acceleration trace, other than the minimal number required to accommodate the required reversals in the movement sequence, tend to cluster around points in the sequence where participants transition from one subsequence to the next. Therefore, zero crossings are indicative of the additional processing costs associated with executing movement subsequences (Panzer & Shea, 2008). The reaction time (RT) data from the secondary task were adjusted by excluding trials with extremely short (<150 ms) or extremely long (>2 SD’s) RT’s. The values of the two individual RT’s were averaged across block. Statistical analyses were computed with SPSS for Windows Version 25.0 (IBM Corp., Armonk, NY, USA).

## RESULTS

**Figure 2:**
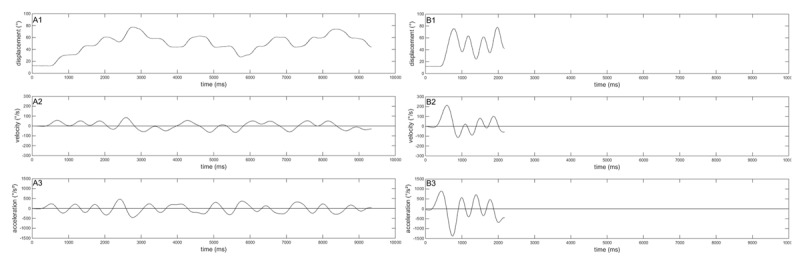
Kinematics of a single participant. Examples of the time series lever position in degree, velocity and acceleration from one participant during acquisition from Trial 3 (A1 to A3) and Trial 150 (B1 to B3).

Examples of kinematics displacements (displacement, velocity, and acceleration) for a single participant during acquisition are displayed in ***Figures 2A*** and ***2B***. In Trial 3 at the beginning of acquisition, the participant responded to targets in what appeared to be discrete steps (A1), dwelling at the target location before initiating a movement to the subsequent target in the sequence. At this stage velocity (A2) and acceleration (A3) values are generally low. By the end of practice (Trial 150), the movement sequence was fluid (B1) with increased velocity (B2) and acceleration (B3).

### ACQUISITION

**Figure 3:**
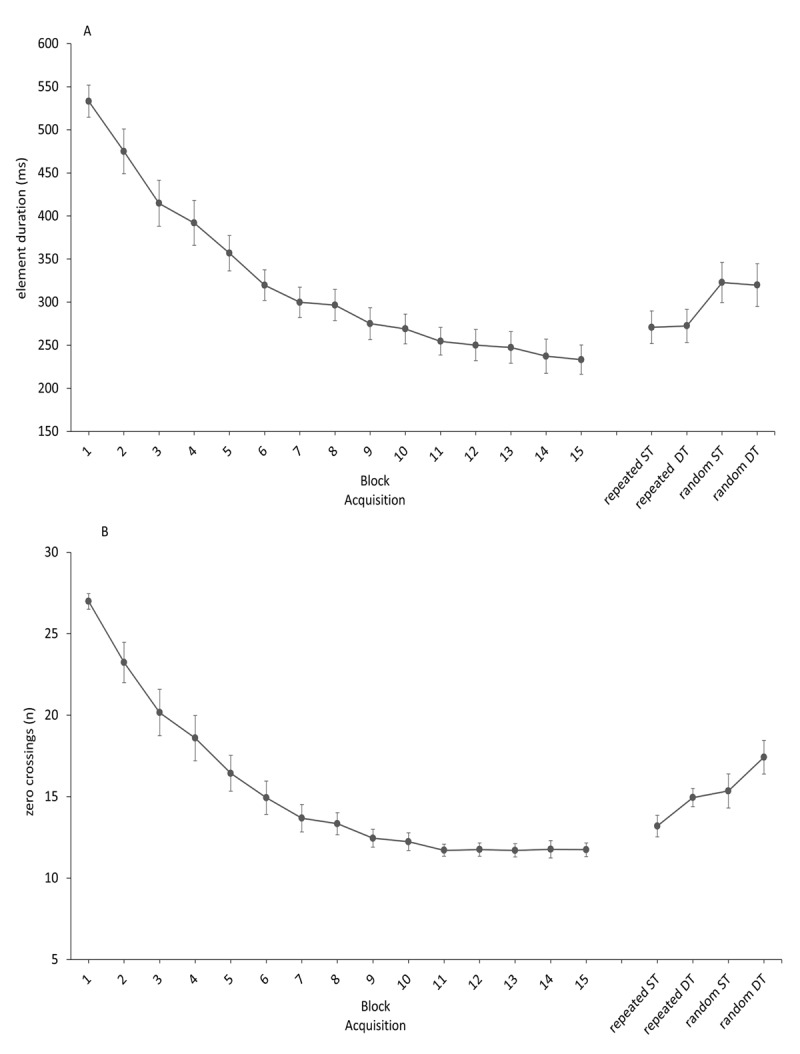
Element duration during acquisition, retention and transfer testing. Element duration and the standard error of the mean (SEM) during acquisition and during retention and transfer tests (3A) and the mean number of zero crossing and SEM during acquisition and during retention and transfer tests (3B). Note, the abbreviation ST is single-task, and DT is dual-task.

**Element duration**: The mean element duration and the standard error of the mean’s (SEM) during acquisition and for the retention and transfer tests are provided in ***Figure 3A***. The analysis of the element duration detected a main effect of Block, *F*(14,210) = 52.92, *p* < .001, *η_p_²* = .78. Duncan’s new multiple range test indicated that total response time decreased through Block 8. No differences were detected for Blocks 9 to 15.

**Zero crossings**: The mean number of zero crossings and the SEM during acquisition and for the retention and transfer tests are provided in ***Figure 3B***. The analysis of the zero crossings detected a main effect of Block, *F*(14,210) = 61.99, *p* < .001, *η_p_²* = .81. Duncan’s new multiple range test indicated that the zero crossings decreased through Block 4. No differences were detected for Blocks 5 to 15.

### RETENTION AND TRANSFER TEST

**Figure 4:**
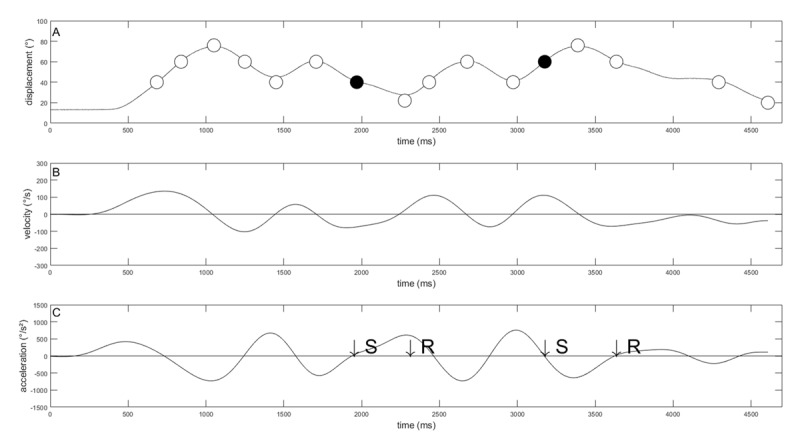
Kinematics of a single participant on the repeated test in a dual-task situation. Examples of the time series lever position in degree (A), velocity (B) and acceleration (C) from one participant on the repeated sequence in a dual-task context. The filled dots in A indicate where the auditory stimuli for the secondary task occurred. The arrows at C provided to demonstrate the impact of the secondary task on the acceleration trace. The abbreviation ‘S’ is for Stimulus, and ‘R’ for response.

An example of kinematic variables on the repeated tests in the dual-task context from a single individual are shown in ***Figure 4***. At the filled dots at ***Figure 4A***, the secondary task occurred. In ***Figure 4C*** the arrows with ‘S’ symbolized where the stimuli (tone) occurred and the arrows with ‘R’ where the response occurred in the acceleration trace.

**Element duration**: The 2 (Sequence: repeated, random) × 2 (Test: single, dual) ANOVA with repeated measures on both factors indicated a main effect of sequence, *F*(1,15) = 29.64, *p* < .001, *η_p_²* = .66. Element duration was slower for the random sequences compared to the repeated. All other analysis failed to reach significance.

**Zero crossings**: The 2 (Sequence: repeated, random) × 2 (Test: single, dual) ANOVA with repeated measures on both factors indicated main effects of Sequence, *F*(1,15) = 18.81, *p* = .001, *η_p_²* = .56, and Test, *F*(1,15) = 27.67, *p* < .001, *η_p_²* = .65. The sequence was performed with a higher number of zero crossings under a dual-task situation compared to a single-task, and the number of zero crossings for the random sequence was higher compared to the repeated sequence.

**Reaction time**: A paired sampled t-test for the repeated and random sequence under the dual-task condition failed to indicate a significant difference for the RT’s between the repeated and the random sequence, *t*(1,15) = –1.82, *p* > .05.

## DISCUSSION

The experiment was designed to determine the impact of a dual-task on the stability of a sequence response structure. A 16-element movement sequence was practiced for 150 trials under a single-task condition. It was predicted that a dual-task would result in increased element duration. This prediction arises from earlier findings, that the response structure essentially determines the speed with which the subsequences are processed and executed and the theoretical assumption that a response structure is represented in an abstract manner and therefore sequence execution requires additional processing demands and attention (e.g. Hikosaka et al., 1999; Shea et al., 2011).

During acquisition the movement sequence was performed faster and smoother as indicated by the decrease in element duration (~300 ms) and in the number of zero crossings (~15). Note that a minimum of 8 zero crossings would be expected, because of the required movement reversals. Additional zero crossings reflect unneeded reversals and temporary lapses in acceleration in an otherwise fluid movement response pattern. Panzer & Shea (2008) have shown that zero crossings, other than the minimal number required to accommodate the required reversals, tend to cluster around points in the sequence where participants transition from one subsequence to the next. Note, zero crossings are indicative of the additional processing costs associated with executing movement subsequences through concatenation. The average number of zero crossings at the end of acquisition was 11.

Learning of the movement sequence was clearly indicated by performance levels observed on the retention and transfer tests on Day 2. The repeated sequence was performed with lower element duration and lower numbers of zero crossings compared to the random sequence. The mean element duration for each element at the random sequence was above 300 ms. This time is sufficient to process response produced feedback information (Keele & Posner, 1968) and to perform each element in a serial and discrete step by step manner. Interestingly the dual-task did not affect the element duration in the repeated sequence. According to previous findings that the sequence structure essentially determines the speed with which the subsequences are processed and executed (Park & Shea, 2005), this finding indicated that following 150 Trials of practice the execution of the subsequences in the repeated sequence was not affected by a dual-task. However, a dual-task during sequence execution increased the number of zero crossings for the repeated sequence. That finding is also illustrated for individual performance in ***Figure 4***. Momentary delays appeared to accrue not only to accommodate the reversal points, they can also be observed between the reversal points. This finding indicated that a dual-task induced additional processing costs by transitioning from one subsequence to the next through concatenation. These findings are only partially consistent with our initial hypothesis and with the theoretical perspective of Hikosaka et al., (1999), who proposed that abstract representations are primarily responsible for sequence execution in the early stages of learning, and at this stage of learning sequence execution requires additional processing costs (Shea et al., 2011) and attention. The current results show that the processes of imposing a structure on a movement sequence with 16 elements reduced the processing demands in the control of the movement speed to execute the sequence, but the concatenation of the subsequences still requires some processing demands. This view is consistent with the parallel processing notion, which suggests that processes, involved in planning the next subsequence, started during the execution of the current subsequence (Kovacs, Muehlbauer, & Shea, 2009). Performing a secondary task during sequence execution increases the processing demands, which results in performance detriments. However, it has been noted that a simple reaction time task is a discrete task, very limited and not very likely to produce severe cognitive interference (Heuer, 1996). While both tasks required similar output modalities motor interference can be accountable for the increased number of zero crossings. The detailed inspection of the individual data in ***Figure 4*** partially corroborated this assumption, because additional zero-crossings occurred near the time the auditory stimuli was presented at Element 7 and Element 12 and another when the motor response was executed for the second auditory stimulus. Of course, a potential limitation of the current experiment is the amount of practice. It is possible that extended practice refines the response structure and that subsequences become inter-associated, by a process that is known as “co-articulation” or “dynamical optimization” (Jordan, 1995) and that a secondary task induces less performance detriments. Clearly, the next step for future research is to tackle the question of delineating the nature of the contribution made through extended practice on the concatenation process and the development of a sequence response structure in a dual-task situation and if additional practice immunized the linkages between the subsequences against a dual-task. The theoretical implication of these findings will be important, because it points attention to more cognitive issues related to response preparation and parallel processing (Kovacs et al., 2009).

## DATA ACCESSIBILITY STATEMENT

Data available at: https://osf.io/gd43y/.
